# Can Drinking Microfiltered Raw Immune Milk From Cows Immunized Against SARS-CoV-2 Provide Short-Term Protection Against COVID-19?

**DOI:** 10.3389/fimmu.2020.01888

**Published:** 2020-07-28

**Authors:** Samir Jawhara

**Affiliations:** ^1^CNRS, UMR 8576 - UGSF - Unité de Glycobiologie Structurale et Fonctionnelle, INSERM U1285, Lille, France; ^2^Medicine Faculty, University of Lille, Lille, France

**Keywords:** SARS-CoV-2, COVID-19, coronavirus, immunoglobulin, IgG, colostrum, immune milk

## Abstract

The emergence of severe acute respiratory syndrome coronavirus 2 (SARS-CoV-2), which causes severe respiratory tract infections in humans (COVID-19), has become a global health concern. Currently, several vaccine candidates against SARS-CoV-2 are in clinical trials but approval of these vaccines is likely to take a long time before they are available for public use. In a previous report, the importance of passive immunity and how immunoglobulin (Ig)G collected from recovered coronavirus patients could help in the protection against COVID-19 and boost the immune system of new patients was reported. Passive immunity by immunoglobulin transfer is a concept employed by most mammals and bovine IgG has a role to play in human therapy. IgG is one of the major components of the immunological activity found in cow's milk and colostrum. Heterologous transfer of passive immunity associated with the consumption of bovine immune milk by humans has been investigated for decades for its immunological activity against infections. This short review focuses on passive immunity and how microfiltered raw immune milk or colostrum collected from cows vaccinated against SARS-CoV-2 could provide short-term protection against SARS-CoV-2 infection in humans and could be used as an option until a vaccine becomes commercially available.

The emergence of severe acute respiratory syndrome coronavirus 2 (SARS-CoV-2), which causes severe respiratory tract infections in humans (COVID-19), has become a global health concern (1, 2). On 30th January 2020, the World Health Organization declared the outbreak of SARS-CoV-2 to be a public health emergency of international concern (3). Most coronaviruses cause animal infections but can evolve into strains that are able to infect humans. Coronaviruses, belonging to the family Coronaviridae, are enveloped viruses with positive-stranded RNA (4) and contain four main structural proteins: spike (S) glycoprotein, envelope (E) protein, membrane (M) protein, and nucleocapsid (N) protein. The entry of coronavirus into host cells is mediated by an envelope-anchored S-glycoprotein, which is responsible for binding to a host receptor and then fusing to viral and host membranes (4). SARS-CoV-2 is believed to be related to bat and pangolin coronaviruses, as shown by genetic analysis that placed this new virus in the genus Betacoronavirus and subgenus Sarbecovirus (lineage B), suggesting that the origin of SARS-CoV-2 is probably bat coronavirus (BatCoV RaTG13) and that pangolins could be a possible intermediate host (5, 6). In terms of the interaction between SARS-CoV-2 and its host, it has been reported that angiotensin converting enzyme 2 (ACE 2) and serine protease TMPRSS2 are used by the S-glycoprotein of SARS-CoV-2 as receptors similar to those of SARS-CoV (7, 8).

Currently, different academic institutions and pharmaceutical companies worldwide have started programs to develop and test vaccine candidates against SARS-CoV-2 in clinical trials. An S-glycoprotein-based vaccine is a promising approach that has attracted the attention of scientists, since S-glycoprotein can be directly recognized by the host's immune system (9). For the first coronavirus (SARS-CoV-1), which was identified in Guangdong province, China, in November 2002, different vaccines were developed and tested in animal models. Some of these vaccines prevented animal infection after challenge with SARS-CoV-1. Kapadia et al. showed that neutralizing antibodies against SARS-CoV-1 could be detected in sera from mice immunized with S-glycoprotein of SARS-CoV-1 (10, 11). Additionally, naïve mice were protected from SARS-CoV-1 infection after the passive transfer of immune sera containing neutralizing antibodies, suggesting that the administration of neutralizing antibodies against SARS-CoV-1 S-glycoprotein is a promising strategy for post-exposure treatment and prophylaxis (10, 11).

## Role of Hyperimmune Plasma-Rich in IgG Collected From Recovered Coronavirus Patients in the Treatment of COVID-19 Patients

In a recent report, I emphasized the importance of immunoglobulin (Ig)G collected from recovered coronavirus patients in protecting against COVID-19 and boosting the immune system of new patients (1). In terms of the clinical evidence on hyperimmune plasma-rich IgG collected from recovered coronavirus patients, two critically ill patients with COVID-19 were treated with convalescent plasma containing SARS-CoV-2 IgG antibodies from recently recovered donors (12). Subsequent to convalescent plasma treatment, lymphocyte counts increased, oxygenation improved, and inflammatory markers decreased in both individuals, indicating that the use of convalescent plasma therapy could potentially improve clinical outcomes (12). In line with the results of this study, the concentration of SARS-CoV-2 IgG antibodies in mild, general, and recovering patients was similar in male and female patients while in patients with severe disease, more female patients compared to males had a relatively high concentration of serum SARS-CoV-2 IgG antibodies (13). This study revealed a discrepancy in SARS-CoV-2 IgG antibody levels between male and female patients, which may be a potential cause of the different outcomes of COVID-19 between the sexes (13). Shen et al. showed that five critically ill patients with COVID-19 and severe pneumonia had rapid progression and a continuously high viral load despite antiviral treatment (14). These patients were treated with convalescent plasma transfusions and had an improvement in their clinical status; in particular, their body temperature normalized within 3 days and viral loads decreased and became negative within 12 days after transfusion (14). This approach of hyperimmune plasma is helpful for newly infected or convalescent patients, but a strategy to boost the immune system against SARS-CoV-2 infection is also crucial since effective and safe vaccines against SARS-CoV-2 are not yet available and approval of these vaccines could take a long time before they become available for public use.

## The Importance of IgG From Colostrum or Cow's Milk in the Elimination of Pathogens

It is well-known that bovine colostrum and cow's milk are highly rich in IgG that binds to many gastrointestinal and respiratory pathogens that infect humans, supporting cross-species activity between bovine and human IgG (15). Cow's milk is accessible to the whole population and drinking microfiltered raw immune milk from cows vaccinated against SARS-CoV-2 (example SARS-CoV-2 S-glycoproteins) could provide individuals with short-term protection against the SARS-CoV-2 infection until vaccines become commercially available. Immunized cows can produce specific IgG antibodies against SARS-CoV-2 glycoproteins in milk or colostrum. Microfiltered raw milk is widely available in French grocery stores and has been commercially successful. The microfiltration process removes all pathogens from the milk without denaturation of proteins or nutritional changes that could be caused by the ultra-high temperature pasteurization of milk (16, 17).

Passive immunity through ingestion of colostrum and bovine milk is essential for the survival of newborn animals. For this purpose, the vaccination of pregnant cows ahead of calving has been shown to be effective in transmitting protection against infections to the newborn calf. In contrast to human milk, in which the principal immunoglobulins are IgA, IgG1, IgG2, and IgM, IgG1 is the major immunoglobulin in cow's milk, in particular in colostrum, whereas IgM, IgA, and IgG2 are present at lower concentrations (18). The concentration of IgG1 is 100-fold higher in colostrum than in cow's milk (19). IgG from non-immunized cows can interact with different types of pathogens, including viruses. Interestingly, bovine IgG can interact with human Fcγ receptors (FcgR), supporting the idea that this interaction can enhance the presentation of antigens to T-cells, as well as phagocytosis by leukocytes (19, 20). An experimental study showed that bovine colostrum increased the proportion of CD8+ T cells post viral challenge in mice (21). IgG also has other functions, including the agglutination of pathogens, complement fixation for pathogen lysis, inhibition of pathogen metabolism by blocking enzymes, and neutralization of viruses. Bovine-colostrum-derived IgG can inhibit the NF-κB signaling pathway as well as the production of pro-inflammatory cytokines in intestinal cells (22).

The vaccination of cows against new pathogens before collecting their milk or colostrum can increase the specificity of IgG in the milk or colostrum and the new pathogen should be neutralized after consumption of hyperimmune colostrum or cow's milk. Different experimental and clinical studies have explored the effect of oral bovine IgG in the prevention or treatment of viral infections, including the use of IgG-derived hyperimmune colostrum or cow's milk (23–26). Hyperimmune bovine IgG can bind directly to the virus, preventing the adhesion of these pathogens to intestinal epithelial cells (25–27). In line with this study, the treatment of mice with immune IgG derived from cows immunized against LPS was associated with an increase in the number of splenic NKT cells, indicating that oral administration of hyperimmune colostrum preparations can alleviate chronic inflammation, including liver injury (28).

## Application of IgG-Derived Hyperimmune Colostrum for Human Health

In terms of the application of IgG-derived hyperimmune colostrum for human health, bovine IgG-derived colostrum has been reported to be resistant to proteolysis, supporting the idea that IgG-derived colostrum contains trypsin inhibitors that promote the survival of these antibodies throughout the gastrointestinal tract (29, 30). The strongest evidence of the protective effects of bovine IgG isolated from colostrum has been found in immunosuppressed HIV patients, who have a decreased capacity to resist infections and are highly susceptible to diarrhea, in particular that induced by *Cryptosporidium, Campylobacter*, and Amoeba (31). In these studies, there was a significant improvement in the management of patients with HIV-associated diarrhea and an increase in body weight and lymphocyte CD4+ counts (31). Odong et al. showed that bovine IgG isolated from colostrum is efficient at boosting the immune response in HIV-positive children, as shown by the significant increase in lymphocyte CD4+ counts concomitant with the enhancement of blood hemoglobin levels and serum albumin (32). Different investigations have reported the beneficial effects of IgG and colostrum on viral respiratory tract infections. Bovine IgG can neutralize respiratory syncytial virus (RSV) *in vitro*, a common childhood pathogen that causes many upper respiratory tract infections in infants (26). Furthermore, dietary bovine colostrum reduced the severity of human (h)RSV infection and enhanced the CD8 T-cell response during hRSV infection (21). IgG and F(ab')2 isolated from hyperimmune colostrum from cows vaccinated with an influenza A vaccine prevented influenza infection in mice infected with a sub-lethal dose of this virus (33). With regard to influenza virus, bovine IgG and colostrum enhanced natural killer cell activity and a lower viral burden was detected in the lungs after the infection of mice with influenza A virus. Additionally, a primary culture of small intestine epithelial cells stimulated with colostrum showed a decreased interleukin-6 production mediated by Toll-like receptor (TLR)-2 and TLR-4 blocking antibodies (34). Bovine colostrum supplementation reduced the severity of viral upper respiratory tract infections in children deficient in IgA (35). The effect of oral administration of colostrum on influenza-associated complications in hospital was explored in patients that received colostrum alone, colostrum combined with vaccination, or vaccination alone in a group of high-risk cardiovascular patients. The colostrum-only group showed significantly lower influenza-associated complications compared to the vaccination only group (36). Additionally, colostrum prevented influenza infection in healthy volunteers at a rate comparable to influenza vaccination (36). Supplementation of IgG1 antibodies from colostrum against bovine coronavirus (BCV) prevented calves from developing viral infections when compared to those treated with low titers of IgG1, showing that IgG1 antibodies from colostrum are crucial for protection against BCV infection (37). Orally-administered egg yolk and colostrum powders from hens and cows vaccinated with inactivated BCV protected calves from coronavirus infection-induced diarrhea in neonates (38).

In conclusion, after the emergence of SARS-CoV-1 in November 2002, several vaccine candidates were developed and tested in animal models and in some clinical trials in humans. However, effective and safe vaccines are not yet commercially available for public use. Nevertheless, the different approaches used in these studies will help to optimize the efficiency of current vaccine candidates against SARS-CoV-2 by inducing a protective immune response and not causing disease. One effective approach has been the passive transfer of immune sera collected from animals immunized with SARS-CoV-1 to naïve animals, conferring protection against subsequent SARS-CoV-1 infection. Based on passive immunity, the vaccination of cows against SARS-CoV-2 before collecting their milk or colostrum may increase the specificity of IgG in the milk or colostrum against this new coronavirus and offer short-term protection to individuals who consume this hyperimmune cow's colostrum or immune milk. In the clinical trial, healthy subjects received human polyclonal IgG antibody (SAB-301) produced from cattle immunized with a MERS coronavirus vaccine and single infusions of this IgG antibody, up to 50 mg/kg, appeared to be safe and well-tolerated in healthy participants (39, 40). In line with this clinical observation, the company SAb Biotherapeutics in South Dakota has genetically modified cows to provide human IgG antibodies against SARS-CoV-2. Currently, this company is planning to start clinical trials of this human IgG against SARS-CoV-2 this summer (2020) (41).

## Author Contributions

SJ drafted this review.

## Conflict of Interest

The author declares that the research was conducted in the absence of any commercial or financial relationships that could be construed as a potential conflict of interest.

## Abbreviations

ACE 2: angiotensin converting enzyme 2
BCV: bovine coronavirus
COVID-19: coronavirus disease 2019
HIV: human immunodeficiency virus
Ig: immunoglobulin
RSV: respiratory syncytial virus
SARS-CoV-2: severe acute respiratory syndrome coronavirus 2
TLR: Toll-like receptor.
